# Hit by the wave: The experiences of adult males living with lymphoedema

**DOI:** 10.1371/journal.pone.0304577

**Published:** 2024-05-31

**Authors:** Josianne Scerri, Sarah Cilia Vincenti, Michael Galea, Carmel Cefai, Paulann Grech, Alexei Sammut, Christie Attard

**Affiliations:** 1 Faculty of Health Sciences, Department of Mental Health, University of Malta, Msida, Malta; 2 Faculty of Health, Science, Social Care and Education, Kingston University, Surrey, United Kingdom; 3 Centre for Resilience and Socio-Emotional Health, University of Malta, Msida, Malta; Sefako Makgatho Health Sciences University, SOUTH AFRICA

## Abstract

Lymphoedema arises when the lymphatic system has been damaged and may occur secondary to cancer treatment. While much of the extant literature focuses on quality of life in females with breast cancer- related lymphoedema, this study explores the impact of living with lymphoedema secondary to cancer treatment in males. Semi-structured interviews were conducted with 13 male participants, aged between 50 to 85 years. Data collected were analysed using interpretative phenomenological analysis. The super-ordinate theme ‘Hit by a wave’ encompasses the profound impact of lymphoedema on the participants’ quality of life. The males expressed body image concerns and struggled with feelings of frustration, anger, anxiety and depression. Physical changes such as weight increase, disrupted sleep, pain, swollen genitals and urinary difficulties were experienced. Changes in lifestyle were also expressed, such as an increased dependence on carers; work-related and role changes. Nevertheless, many participants endorsed the need to focus on the present moment and expressed a heightened appreciation of authenticity in life. By understanding the unique narratives of males with lymphoedema, health care practitioners together with patients can formulate care plans that truly resonate with the needs, concerns, and experiences of males living with lymphoedema.

## Introduction

Lymphoedema refers to tissue swelling in an affected body part that develops from damage to the lymphatic system [1], and can arise secondary to cancer treatment, such as lymph node surgery or radiotherapy. Reported prevalence rates of lymphoedema following prostate cancer treatment are as high as 20% [2]. Nevertheless, there is consensus that such rates are probably underreported, as many cases go unrecognised or undiagnosed [3–5]. The gold standard of lymphoedema management is complete decongestive therapy, which comprises manual lymphatic drainage, exercise, skin care and the use of compression garments [6].

The rise in cancer survival rates, coupled with an increased life expectancy in developed countries, emphasises the need to focus more attention on the impact of living with lymphoedema [7]. Several studies have explored the experiences of persons having lymphoedema, with the majority of these studies targeting breast cancer related lymphoedema (BCRL) and quality of life (QoL) in female patients [e.g., 8,9]. Nonetheless, it is important to explore the lived experiences of males with lymphoedema, as various quantitative studies have identified differences in the impact of lymphoedema by gender. For instance, a prospective, study exploring coping efficacy for lymphoedema among 277 melanoma patients, demonstrated that males were found to have poorer coping efficacy scores than females [10]. Whilst a study examining the impact of chronic lower limb oedema in a sample of 122 participants, identified that females reported having a better QoL than their male counterparts [11].

Corresponding qualitative studies also follow the same trend, with the majority targeting breast cancer patients having lymphoedema. Nonetheless, studies exploring specific lived experiences have been conducted, such as: undergoing compression bandaging [12]; (ii) accessing lymphoedema clinics [5]; (iii) renegotiating hope [4]; (iv) work experiences [1] and (v) the impact of surgery on QoL [6]. The overarching narrative of these qualitative studies target the experience of living with lymphoedema, predominantly from a female perspective. They underscore the debilitating physical effects of lymphoedema namely pain, swollen limbs, skin prone to uncontrollable weeping and sleep disruption [5]. Living with lymphoedema also triggered altered body image concerns and served as a perpetual reminder of a potentially deteriorating cancer condition [13]. The participants also experienced a loss of spontaneity, since the constant self-management of their condition required meticulous planning, often described as time-consuming, exhausting, and frustrating [4,5]. Family relationships were often affected, since patients became dependent on family members [14], whilst disengagement from leisure activities precipitated a loss of social relationships [13]. Work-related and financial implications were identified, with reported changes in work dynamics, such as a reduction/termination of employment and renegotiation of their work role [1,6]. Having lymphoedema also posed a threat to the sense of masculinity in males with prostate cancer, by impacting their identity, productivity and sexual functioning [15].

The current study addresses the gap in extant literature by providing an in-depth account of the lived experiences of males living with lymphoedema, secondary to cancer treatment.

## Methods

### Research design

The qualitative design chosen was interpretative Phenomenological Analysis (IPA) as it enables an in-depth exploration of the participant’s lived experiences, whilst acknowledging the researcher’s interpretative role [16]. The IPA design is based on three theoretical underpinnings: phenomenology, hermeneutics and ideography. Phenomenology aims to describe the lived experiences of the participants from their subjective first-person accounts, whilst hermeneutics is concerned with the analyst striving to understand the participants’ sense making [17]. The ideographic nature of the present study is represented in the detailed analysis of each case, before making cross comparisons between the different cases.

### Participants

The recruitment period for this study was between 9th October 2021-15^th^ March 2022. Purposive sampling was used to recruit thirteen male participants having lymphoedema. The inclusion criteria applied were adult males being treated for lymphoedema, secondary to a cancer diagnosis. Most participants had prostate cancer (n = 6), followed by sarcoma (n = 4), testicular cancer (n = 1), breast cancer (n = 1) and melanoma (n = 1) respectively. The mean age of participants was 68.6 years, with an age range varying between 50 to 85 years.

### Data collection

Semi-structured audio-recorded interviews were conducted by the first author [JS]. The participants were asked the following open-ended question namely: “can you describe your experience of living with lymphoedema?”. When additional information was required probing questions such as ‘how did such a situation affect you?’ were utilised. The duration of the interviews ranged between 40–90 minutes and they were transcribed verbatim.

### Ethical considerations

The study was conducted in accordance with the Declaration of Helsinki and approved by the Ethics Research Committee of the Faculty of Health Sciences, University of Malta [No: 3915_24122019]. Potential participants were provided with an information sheet by an intermediary (physiotherapist) informing them about the nature of the study. Persons who expressed their willingness to participate with the intermediary, were then contacted by the first author (JS). A meeting was held with the participant and they were provided with an opportunity to clarify any queries prior to consenting. A consent form was then signed, indicating voluntary participation in the research.

### Data analysis

Data analysis adhered to Smith et al.’s [16] guidelines. This commenced with a case by case reading and re-reading of the transcript. Throughout this process, exploratory statements were made for each case, that focused on descriptive, linguistic, and conceptual insights. Extracts were coded with one or more emergent themes being identified. Connections across the various emergent themes were then identified, to determine how they related to each other. Those themes that represented similar understandings were grouped under a single super-ordinate theme. The superordinate theme and themes were then formulated into a narrative account supported by participant quotes.

### Trustworthiness

The model by Yardley [18] was used to evaluate trustworthiness. This model incorporates four aspects namely: sensitivity to context; commitment and rigour; coherence and transparency and impact and importance. Sensitivity to context was achieved by integrating relevant literature and by the inclusion of verbatim extracts. Commitment and rigour were ensured by providing details on the research design, participant recruitment and data analysis. Coherence and transparency were enabled by providing a rationale for decisions taken and by aligning the present research to the underlying theoretical assumption of IPA. A reflexive diary was utilised to highlight any biases and observations, thus enhancing transparency. The principle of impact and importance was addressed by exploring an understudied topic, namely the experiences of males living with lymphoedema.

## Results

The analysis conducted led to the extraction of one super-ordinate theme ‘Hit by the Wave’, that represented the overwhelming impact that living with lymphoedema had on male participants. Additionally, the five themes presented in Table 1 encapsulate the quality-of -life domains that were impacted by living with lymphoedema namely the ‘physical’, the ‘functional’, the ‘psychoemotional’, the ‘social’ and the ‘spiritual’ domains. Verbatim extracts are provided to further illustrate each theme.

**Table 1:** Thematic representations relating to the experiences of males living with lymphoedema.

Super-ordinate theme	Themes	Subthemes
Hit by the Wave	Having a balloon shaped body part	Pain Burning sensation Itching Urinary difficulties Sleep disturbance Weight gain
	A changed Lifestyle	Work related Role change Dependence on others Reduced flexibility
	Feeling never out of the woods	Frustration Anxiety at impending doom Depressed Anger Repulsion at body image Upset
	Social impact	Withdrawal from social activities Altered family dynamics Changes in attire for social inclusion Social support
	Searching for meaning	Feeling forsaken by God Living in the moment Grateful mindset Being authentic Appreciative of life events

### Having a balloon shaped body part

Participants referred to the physical impact of lymphoedema. Swollen body parts were described using terms such as a ‘balloon’ or ‘an elephant’s leg’. Having a swollen body part had various implications, such as weight gain and consequently ill-fitting clothing. One participant (Frank) described wearing a trousers with the fabric stretched tight over one leg and loose over the other.

*I had the leg of an elephant…so on one leg the fabric fitted tightly compared to the other leg* (Frank)

Michael, another participant, voiced his struggles to urinate due to a swollen genital area. This preoccupied him as he envisaged a future need for a catheter to urinate. The use of a catheter alarmed him, as he perceived it as a very ‘unnatural way’ to remove urine from the body.

as a result of this [swelling], my penis is affected, I can pass water, but you know physically it’s a bit difficult to do so. And the skin there has been affected by the lymphoedema. And this is worrying because if it gets worse, I may be eventually unable to pass water in this way and may have to have a surgical intervention to allow me to do so, you know externally. (Michael)

This accumulation of excess fluid in body parts, at times required removal with a syringe. Andrew equated the fluid to a substance that was ‘alien’ to his body and that had to be eliminated. He further compared the dull coloured fluid to something dead and consequently experienced relief when it was removed.

Imagine seeing the syringe filling up with the colour of something dead, something alien to you. That fluid was in you and that affects you, but at the same time you feel satisfied that it [the fluid] is now out (Andrew)

Some participants articulated experiencing intense burning and itching in swollen body parts. The relentless pain was so overpowering that painkillers were required, and some previously enjoyable activities had to be avoided. For one participant (Karl) the pain was so unrelenting, that he contemplated death as an escape and end to his misery. The following excerpt also illustrates how the dimensions of wellness (in this case the physical and the psychological) were interlinked.

A*fter my third chemo session my genitals also became swollen*, *and with further chemo sessions the lymphoedema increased*, *I struggled with itching*, *swelling along my leg and tightening of my skin with a burning sensation*. *It felt like having a stove attached to both of my legs burning all the time… my morale*, *my psyche broke down*, *I used to pray*, *I asked God to intervene and to hasten my death because I just could not tolerate what I had to bear*. *(Karl)*

Within this scenario, the participants strived to exert some control over the discomfort experienced. They described searching for causal triggers that aggravated their lymphoedema. They identified both a poor posture and/or lack of mobility and targeted these aspects to control the pain experienced.

*I couldn’t sit down [in the car]; it was so painful*, *and my leg felt heavier [due to fluid retention]*. *We used to go out by car*, *and I would tell them*: *‘stop*, *stop I can’t take it anymore’*. *I used to then go out and walk a bit for some relief*. (Joseph)

### A changed lifestyle

For the thirteen participants, the functional impact of lymphoedema had a considerable impact on their overall quality-of-life. Their altered functional ability was concerning as it directly affected their employment and even breadwinner status in some cases. For instance, Adam explained that he changed his job to one that was less strenuous and challenging.

*I used to work as a purchasing officer and storekeeper in various supermarkets and hotels*. *Obviously*, *I had to stop working after the cancer diagnosis*, *it was a hard and tiring job which I could no longer do*. *Then I just switched to the part-time job that I previously had with a sports agency* (Adam)

Routine tasks such as drying off after a shower or wearing socks were now considered as monumental tasks. In these circumstances, most males expressed becoming reliant on others, turning to them for assistance.

*My wife has to help me*, *otherwise I can’t put it [the sock] on*, *on my own*. *It requires certain movements on my part*, *in fact when I bend over it gets very*, *very painful*. (Chris)

For some participants, the necessity to request help from others aroused profound resentment. This dependence on others conflicted with their identity of being an independent person. It also posed a problem for those participants who lived alone and who could not request help with various routine tasks. One participant, Stephen elaborated on a strategy to increase flexibility in the limb by improving circulation to that area. He described striving to cope by walking around the room prior to any attempts to put on a sock.

*I’m a person who happily prided himself on being independent*, *I kind of resent having to trouble anybody*. *I don’t really like to feel as though someone was acting as my personal servant*. *For example*, *to get a sock over the top of my foot*, *I would have to walk around the apartment for fifteen to twenty minutes to get enough flexibility in my knee and then attempt to put the sock on*. (Steven)

Mixed emotions were expressed by participants relating to the heavy bandaging around an affected limb. The males acknowledged such bandaging as necessary, yet the reduced flexibility imposed various functional implications. Several males commented that tasks requiring adequate control of limbs such as driving, had to be avoided.

*when I go for Physiotherapy they wrap my leg in bandages*, *and they are intentionally pulled tight and so I can’t do certain movements*. *My fear is that since my leg is very heavy*, *I may press the gas pedal and I will accelerate more than I should [with the car]*. (Chris)

### Feeling never out of the woods

The experience of lymphoedema profoundly affected participants both psychologically and emotionally. The participants elaborated about the mental distress experienced when some professionals failed to recognize lymphoedema immediately.

*What happened [being diagnosed with cancer] led to another [having lymphoedema] after chemo and they could not find out what it was*, *I mean*, *everybody was sending us here and there*, *we go to one hospital and then they [health professionals] sent us to the ulcer clinic in another hospital* (Karl)

Although the wearing of compression garments was interpreted as beneficial, they posed various challenges. One participant, Kevin described himself as a ‘living carnival in summer’. He explained that wearing shorts alongside a dark compression sock made him conspicuous even in a crowd. Another participant (Paul) emphasised the challenge of wearing a tight compression garment that was unbearable in hot, summer months.

*What happened to you*?*’ people ask me*. *In winter*, *if you wear a trousers or tracksuit the compression garment is covered*. *There is also this elastic band that can be stretched but it is made of a material that is bothersome and not comfortable to wear in summer*. *In winter the temperatures are cooler so you can cope*, *but I don’t know how I will manage in summer even though I’m aware that it does help to decrease the swelling*. (Paul)

For Adam the wearing of compression garments under a trousers, was a strange experience and posed a particular challenge for males. He likened the wearing of a compression garment to the experiences normally encountered by females when wearing tights.

My compression socks keep falling and I feel like a woman constantly raising her tights. At home where I spend a lot of time, this is not a problem however when you go out and are wearing trousers it is challenging to stay raising it [the sock]. (Adam)

Mixed emotions were also expressed about self-management activities. Several participants described the daily exercise routine as time consuming and cumbersome yet acknowledged its necessity in maintaining their overall well-being.

*it’s because it [exercise regimen] takes a lot of time*, *a good twenty to twenty-five minutes*, *and the fact that you must do them whether you like it or not that really bothers me… If you could do them once or twice a week*, *but the fact that they must be done daily is frustrating*, *however they are needed*. (Isaac)

The participants also highlighted scenarios that triggered dread and apprehension such as witnessing more severe cases of lymphoedema. These experiences caused them to fear the impact of lymphoedema on their quality-of life, should their situation aggravate further.

*I worry most about the future*. *Sometimes I surf the internet searching about lymphoedema and I see images of persons with deformed legs*. *Even last time at hospital there was an unfortunate patient with a tremendous swelling in her calf and knee area*, *it was terribly swollen*, *and I imagined what if that had to happen to me*. *It would impede me from leaving the house*. (Isaac)

Living with lymphoedema also triggered feelings of resentment and loss of identity, especially when it impacted the participants’ occupational roles. For many of these males, their identities revolved around their ability to complete various physical tasks. When this ability was compromised (due to having lymphoedema), a sense of powerlessness and anger ensued.

*sometimes that anger consumes me… it is because I want to do some things and I can’t*, *for example I find it very challenging to climb up a ladder*. (Chris)

The magnitude of the psychological and emotional impact experienced, further triggered past addictions in two participants and psychological assistance was required.

after I had these fears, I started smoking again. I had this internal struggle, I knew smoking would not make me feel better, but I was craving smoking for comfort, I had this fear, I then spent over a year being supported by a psychologist. (Andrew)

The psychoemotional impact of living with lymphoedema was also interpreted through age- related expectations. For instance, Karl was upset that he required support appliances for mobility challenges, as these were typically associated with older age adults.

*I used to tell myself is it possible that I have ended up in this state [dependant on using crutches]*? *I am so young compared to others; then why should I have to resort to using crutches*? *Psychologically this really impacted me*. (Karl)

### Social impact

Several participants expressed that they could not maintain various social commitments due to the physical and psychological burden of lymphoedema.

*To get to church you need to climb up a number of steps*. *There is the staircase leading up to the bridge*, *then the steps from the bridge up to the church*, *this discouraged me to manoeuvre*. *So*, *I used to end up following mass from home on Sundays*. (Chris)

Some participants expressed feeling self-conscious about their physical appearance. In fact, they described wearing trousers, to conceal swollen body parts or compression garments. This made them feel more at ease to socialise.

*I like wearing shorts in summer*, *however due to this problem [swollen leg] I have to wear a trousers*. *Obviously*, *I don’t blame people for staring if they see someone in my state*. (Chris)

Shifts in family dynamics were also expressed by most participants. They described that family members, particularly spouses and adult children became overprotective, preventing them from engaging in activities. This scenario reinforced a situation where persons with lymphoedema became increasingly dependent on their informal carers.

*My daughter sort of thinks that I cannot do certain things*. *I own some apartments and I used to do some maintenance work occasionally*. *Currently*, *I have stopped*, *as my daughter does not want me to do anything*. *She insists that she does the work herself*. (David)

Yet for some men, the emotional and practical support provided by family and close friends was pivotal in enabling them to survive the ordeal of cancer and lymphoedema. In the following excerpt, Andrew praised the emotional sustenance that his wife provided:

*During my experience with cancer*, *I was fortunate*, *my wife played an immense role because she was like a sponge and absorbed all the emotions and she had a very positive attitude with me*. *Most of my emotional strength was coming from her and a very few close friends*, *but mainly it was my wife*. (Andrew)

Yet two participants who were not in a stable relationship, preferred sharing intimate details, such as difficulties to urinate with a male friend. For instance, David revealed that living with lymphoedema was a personal matter. Hence, he preferred to share his experiences with a close male friend, rather than with the woman that he was currently dating.

*Yes*, *I would rather share my experiences with a good male friend*. *With women not so much*, *I’m telling you I used to date a woman and I was not feeling comfortable disclosing to her certain things that were happening*…*they are personal* (David)

Interacting with other men having lymphoedema served to encourage them to adhere to a treatment regimen. They described challenging each other when performing exercises recommended by the physiotherapists. However, these interactions also helped the participants to cement friendships amongst themselves.

*we used to meet every Saturday morning*, *then we started meeting on Saturdays and Wednesdays and we used to end up*, *well you know a group of men*. *For example*, *one would ask whether another managed to do six push-ups*, *and the other would state that the last push-up did not count*!! *So*, *in that way*, *you laugh*, *and you have fun*. *Then we would go for coffee*, *and we started building friendships*. (Andrew)

The added benefit of living in a closely-knit social community was endorsed by some participants as providing them with the necessary assistance and support required.

*There are one or two friends who will come and take me out to shop*, *well*, *I only need that help maybe once a week*, *once a fortnight*. *I have a lovely*, *local*, *greengrocer and someone will buy my greengroceries once a week from him*, *and at the end of the day he might even bring them himself*. (Michael)

### Searching for meaning

Most participants reported that their experience of living with lymphoedema impinged on their religious beliefs and/or their outlook to life. One participant vividly communicated how he felt forsaken by God and questioned why this experience had befallen him:

*I used to feel forgotten by God*, *why had he thrown me in this misery*? *Sort of*, *I will be honest*, *I took it all out against Him*, *not intentionally*, *but kind of it was beyond my control*, *out of all these people*, *I used to say but why did he allow this to happen to me*? (Karl)

However, there were many instances where the participants reflected on their illness trajectory, initially with cancer and now with lymphoedema. They reflected on the transient nature of human existence and how a life can change abruptly. This made them appreciate the need to live and focus on the present moment.

*One cannot take things for granted because as human beings we are prone to these things and your life can change from dusk till dawn*, *I would say as quick as a flash*. (Karl)

This appreciation of the fleeting quality of life also triggered Adam’s decision to be his authentic self when engaging with people or situations. Following his experience of living with lymphoedema, he became better aware that time is precious and should be used wisely.

*before I used to be cautious before talking to people*, *now I politely and prudently express whatever I need to*, *because I want to keep enjoying my wife*, *my family and my friends and I do not have to waste my time*, *so I am upfront about who I am*. (Adam)

Another participant (Jesmond) expressed that he developed a grateful mindset after passing through the adversity of an illness. Although he acknowledged struggling with lymphoedema the other alternative, basically ending up dead due to cancer was far worse.

*I think we sometimes forget the good things and we take them for granted and we end up being negative and critical*. *We don’t necessarily think*, *but I’m alive*! *I could have been dead with the cancer*! (Jesmond)

Greater sensitivity or emotional depth was reported by another participant. In the following excerpt, Andrew hinted that as a male he had originally perceived such traits as a sign of weakness. However, these thoughts have now been reframed as an evolved appreciation of life.

*if I am watching television*, *if I am watching a film and there is some romantic moment*, *I started feeling much more emotionally than when I previously watched these scenes*. *I am more emotionally appreciative*, *it’s hitting me much deeper*, *and emotionally I will not say I am weak*, *because I am not weak*, *but I started appreciating other aspects of life more*. (Andrew)

Experiencing lymphoedema has also spurred some males to take an active role in inspiring others who may be struggling with this condition. They also became more vociferous regarding male-specific patient needs and concerns relating to cancer and lymphoedema.

*So I went there*, *I mean in this school hall full of people with the thought that I could perhaps through my experience help someone else*. *I just went through it*, *gave as much information as I could and from the perspective of a man*. (Andrew)

## Discussion

To date extant literature tends to focus on the lived experiences of females having lymphoedema secondary to breast cancer treatment. This study contributes by exploring the experiences of males living with lymphoedema secondary to cancer treatment. The following sections interpret the findings within the context of the Common-sense model as posited by Leventhal et al. [19]. This model postulates that individuals strive to make sense of their experience with a disease and consequently manage the health threat encountered (i.e., lymphoedema post-cancer treatment). This process involves the parallel processing of cognitive and emotional representations that shape the individual’s illness appraisal and management. The cognitive representations discussed in the following section, encompasses the following domains: consequences (e.g., perceived severity and impact of functioning due to lymphoedema); causal triggers; identity (e.g., symptoms of lymphoedema); control and cure perceptions.

### Cognitive illness representations

The present study highlights the profound and multifaceted consequences of lymphoedema, secondary to cancer treatment in males. This finding has implications, as the experiencing of greater consequences of an illness is associated with poorer psychological well-being, social and role functioning [20]. In fact, male participants described that living with lymphoedema had an influence on their physical, psychoemotional, functional, social and spiritual domains. The participants also expressed that living with lymphoedema prevented them from fulfilling ‘traditional’ male roles, such as undertaking maintenance work at home or being a breadwinner. This perceived inability to align with a masculine gender role is linked to poorer psychological health and a lower likelihood of confiding in family or friends following a stressful event [21]. Indeed, some participants expressed that intimate details about their lived experiences with lymphoedema were only shared with a close male friend and/or a person with whom they shared a stable relationship. This may be attributed to discomfort in disclosing intimate details about body parts affected by the lymphoedema. However, it may also align with the traditional male gender role in the Western world, namely that of displaying stoicism and autonomy even in the face of adversity [22]. The experience of living with lymphoedema was also further interpreted from an age- related perspective. In fact, one male participant in mid-adulthood resented having to utilise mobility structures often associated with older adults. This finding further highlights the need for research targeting persons with lymphoedema in early adulthood, as study participants were all in the mid or late adulthood phase.

The causal domain in the common-sense model was also exemplified with participants attributing lymphoedema to the cancer treatment. According to Janoff-Bulman et al. [23] such searches for causal attributes pertaining to symptoms, enables individuals to gain mastery over a situation and give meaning to an illness. The male participants also made downward comparisons when comparing their current experience of living with lymphoedema, to persons having cancer or to persons presenting as more severe cases of lymphoedema. The comparison of one’s current health status (i.e., having lymphoedema) to another severe illness (i.e., having cancer) may have a positive effect on the person as they feel fortunate [24]. Conversely, comparisons to persons having the *same* condition (i.e., lymphoedema) but to a more severe degree, may have a negative effect on the participants, who fear an aggravation of their health status with consequent repercussions.

Participant narratives also frequently targeted another cognitive domain, that of control. Although the self-management of lymphoedema was endorsed as a necessity by study participants, yet a study by Morgan et al. [12] reports that males are less likely to comply with lymphoedema treatment than women. In fact, study participants perceived the need to self-manage as a negative consequence of lymphoedema affecting their overall well-being. They also highlighted their continuous struggles to manage their lymphoedema. Nonetheless, as the self-management of lymphoedema is contingent on feelings of self-efficacy [10], there is a need for males to appraise self-management as a ‘control’ of lymphoedema rather than a ‘consequence’. Health professionals should also further explore and understand the narratives of males when addressing self- management challenges. This would enable the targeting of their needs and concerns that impact on their adherence to treatment. For instance, most study participants highlighted the opportunity to meet up with other males during physiotherapy sessions as empowering. These sessions were perceived as providing them with opportunities for friendly competition and humorous exchanges. This engagement in humorous banter, and challenging others, whilst performing an activity serves to create team solidarity, but also as a means to transmit and perform masculinity [25].

### Emotional illness representations

According to the Common-sense model, whilst persons are cognitively processing a health threat, they are also engaging in emotional processing [19]. The emotional responses to the health threat (such as lymphoedema) are influenced by the unique perceptions of patients to their illness [26], in this case living with lymphoedema. Study participants voiced a gamut of emotions that included fear and anxiety at the thought of cancer returning and/or lymphoedema worsening, to shame and resentment at their altered identity and body-image. The experiencing of such emotions is reported to be positively correlated to anxious preoccupation [27]. Furthermore, although the experience of self-consciousness, shame, and anxiety were voiced in various studies [e.g., 11], the present study contributes by providing an in-depth exploration of the emotional perspective of males with lymphoedema. Male participants also expressed frustration at being dependent on others, such as spouses and/or close friends, even for basic tasks. Wives and daughters were often described as being both an emotional sustenance but also at times, overprotective by hindering them from performing routine activities for fear of injury.

Males also expressed frustration at wearing a compression garment, especially during hot summer months. Two males interpreted the wearing of such garments as incompatible with masculinity, as it was compared to a ‘female experience’ of wearing tights. Hence, the use of compression garments was perceived by some participants as more challenging for males to accept.

Searching the internet for information was also described by several participants. This strategy has become increasingly popular due to its accessibility at any time of day and the immediate provision of continuously updated information [28]. Despite this, the participants expressed that information and images of persons severely impacted by lymphoedema, triggered fear and anxiety, as they envisaged themselves in a similar situation. Health professionals in such scenarios can support males with lymphoedema, by engaging authentically with them and exploring their narratives, that includes their concerns, beliefs and values [29]. This personalized information can then further be negotiated and integrated in any care plans formulated together with the person having lymphoedema.

### Limitations

As typical of IPA research, this study was conducted on a small sample of participants, hence, the findings cannot be generalised to a wider population. However, the aim of the study was not to generalise the findings, but rather to provide an in-depth exploration of the experiences of males living with lymphoedema. Additionally, telephone interviews with participants were conducted since data collection coincided with Covid-19 related restrictions imposed by health authorities. Face-to-face interviews may have fostered more openness and candour.

## Conclusions

The study was designed in response to the underrepresentation of males in lymphoedema research. The impact of lymphoedema on the male participants was profound and multifaceted. Hence, health professionals need to explore the narratives of males living with lymphoedema and address their unique needs and concerns, Furthermore, professionals need to gain a better understanding of the impact of traditional male gender roles on the experiences of males with lymphoedema, such as in relation to help seeking behaviour. There is also a need for lymphoedema services that specifically target the needs of men and for more research among this population such as in relation to the experiences of males in the early adulthood phase that experience lymphoedema.
